# Four-Wavelength Thermal Imaging for High-Energy-Density Industrial Processes

**DOI:** 10.3390/jimaging11060176

**Published:** 2025-05-27

**Authors:** Alexey Bykov, Anastasia Zolotukhina, Mikhail Poliakov, Andrey Belykh, Roman Asyutin, Anastasiia Korneeva, Vladislav Batshev, Demid Khokhlov

**Affiliations:** 1 Scientific and Technological Centre of Unique Instrumentation, Russian Academy of Sciences, 15 Butlerova, 117342 Moscow, Russia; bykov@ntcup.ru (A.B.);; 2 National Research University “Moscow Power Engineering Institute”, 14-1 Krasnokazarmennaya, 111250 Moscow, Russia

**Keywords:** high-energy density beam advanced machining, additive manufacturing, non-destructive evaluation, thermal imaging, spectral imaging, in situ monitoring, multispectral camera, camera calibration

## Abstract

Multispectral imaging technology holds significant promise in the field of thermal imaging applications, primarily due to its unique ability to provide comprehensive two-dimensional spectral data distributions without the need for any form of scanning. This paper focuses on the development of an accessible basic design concept and a method for estimating temperature maps using a four-channel spectral imaging system. The research examines key design considerations and establishes a workflow for data correction and processing. It involves preliminary camera calibration procedures, which are essential for accurately assessing and compensating for the characteristic properties of optical elements and image sensors. The developed method is validated through testing using a blackbody source, demonstrating a mean relative temperature error of 1%. Practical application of the method is demonstrated through temperature mapping of a tungsten lamp filament. Experiments demonstrated the capability of the developed multispectral camera to detect and visualize non-uniform temperature distributions and localized temperature deviations with sufficient spatial resolution.

## 1. Introduction

Recent advancements in high-energy-density (HED) beam advanced machining and metal additive manufacturing (AM) have provided the ability to create parts with unique properties that are unavailable to traditional manufacturing [1,2,3,4,5,6], including the production of complex structures with high accuracy and significant weight reduction. Crucial challenges in the quality control of metal AM technologies are related to in situ monitoring, due to a lack of information about defect formation, which affects the reliability and repeatability of the manufacturing process [7,8].

Thermal imaging of the processing zone addresses the task of in situ temperature monitoring. The basic approach to temperature monitoring in HED industrial processes is point temperature measurement using a pyrometer [2,9,10]. To achieve temperature gradient mapping, one should proceed with thermal imaging. Infrared (IR) radiometric imaging pyrometry [11,12] demonstrates moderate spatial resolution due to larger wavelengths [13] and requires a thorough calibration process to account for the spectral and temperature dependences of material emissivity and gas absorption in the operational area [14,15]. To overcome the need for prior knowledge of emissivity, spectral imaging techniques can be used [12,13,16,17]. The most common spectral thermal imaging techniques include two-wavelength imaging pyrometry (TWIP) and multispectral (MS) and hyperspectral imaging. TWIP provides good temperature measurement uncertainty for high-temperature processes in metal AM [18] and may be achieved by combining red and green color channels of an RGB camera [19]. Despite the relative simplicity and cost effectiveness this technique demonstrates significant temperature uncertainty in the 1000–2000 °C temperature range. Hyperspectral imaging techniques show promise for achieving lower measurement error and eliminating difficulties associated with emissivity dependencies by obtaining the detailed spectra [20,21], while requiring the spatial scanning to acquire a two-dimensional temperature map. MS imaging appears to be an intermediate technique providing two-dimensional images in multiple spectral bands without spatial scanning [22,23].

One of the simplest solutions within the variety of MS camera layouts [24] is the multiaperture filtered camera (MAFC) [25]. It offers relatively high spatial resolution and additional spectral data to enhance the precision of temperature estimation. Meanwhile, this layout maintains the availability of interchangeable spectral filters, while being versatile and offering an adjustable wavelength set to measure spectral intensities distributed along the monotonically increasing part of the Planck curve. This feature provides operational flexibility in dealing with the different observed object temperatures. Since the image sensor (IS) of the MAFC is virtually divided into zones corresponding to the spectral bands, the mentioned MS camera layout involves a specific optical system design. In this work, we describe a basic design concept for an MS MAFC and a general data processing workflow that accounts for the intrinsic properties and residual image distortions inherent to the chosen camera elements before temperature calculation. To provide good flexibility of component choice, the camera’s basic design concept is based on the use of interchangeable, commercially available machine vision lenses equipped with bandpass filters and a custom mirror alignment system. The four-wavelength pixel-wise temperature calculation approach allows for initial temperature estimation with two wavelengths and subsequent fitting of four measured spectral intensities to the theoretical Planck curve by optimizing both temperature and emissivity. To demonstrate the feasibility of the proposed basic design and methods, we developed a prototype of a versatile MS MAFC for thermal imaging applications and experimentally assessed its performance with common thermal imaging test objects.

## 2. Materials and Methods

### 2.1. Experimental Setup

#### 2.1.1. Multispectral Camera

Our MS camera design is developed within the general MAFC layout. Essentially, the MAFC generates distinct images in individual spectral channels using an array of lenses that is compact enough to be positioned in front of the IS [24,26]. The small size of the individual channel components (lenses and filters) presents significant technological challenges. Adjusting the image scale and working distance necessitates the use of additional objective lenses, and reconfiguring the spectral bands requires disassembling the camera. We present a basic design suitable for finite-conjugate imaging with the MAFC using interchangeable commercially available machine vision lenses and bandpass filters.

The use of the proposed MS MAFC in thermal imaging applications imposes certain limitations on its use at finite working distances due to the mutual parallax of each spectral channel’s objectives. The presence of parallax causes each spectral channel to capture individual scenes that only partially overlap, significantly reducing the effective field of view of the MS camera. The obvious solution is to create a hinged structure that allows the objectives to tilt toward each other, enabling each channel to record the same scene without parallax. However, this solution has several design-related limitations. The aperture ratio of each spectral channel’s objectives depends on their diameter, while the resulting field of view depends on their focal length. Additionally, both the IS and the printed circuit board on which it is mounted have finite dimensions, further limiting the placement and size of the objectives. Therefore, it is essential to select the optimal balance between focal length, aperture ratio, and IS size to design an MS camera capable of capturing objects located at both finite and infinite distances. Based on the above considerations, the selection of objectives and IS was made to achieve the desired image scale at a specific working distance, in accordance with the formulas of geometric optics [27]:

(1) $$\beta =\frac{y^{\prime }}{y}=\frac{a^{\prime }}{a^{\prime }}$$

where β is the linear magnification of the objective of each spectral channel, $y^{\prime }$ is the image size of each spectral channel, y is the object size (linear field of view), $a^{\prime }$ is the distance from the objective to the object (working distance), a is the distance from the objective to the IS.

(2) $$f^{\prime }=\frac{aa^{\prime }}{a-a^{\prime }}\;,$$

where $f^{\prime }$ is the focal length of the objective of each spectral channel.

Since the MS camera was developed specifically for thermal imaging applications in AM and HED processes, we selected a linear field of view ranging from 5 to 10 mm, with a minimum working distance of approximately 300 mm for each spectral channel. The camera’s design prioritizes simplicity in manufacturing mirrors and fasteners, as well as ease of assembly and alignment. To achieve this, the IS size needs to be maximized. Based on these parameters, we performed calculations using Equations (1) and (2) and determined that objectives with a focal length of 75 mm were optimal. Subsequent 3D modeling of the MS camera helped us to establish the required objective diameters to eliminate parallax at the specified minimum working distance. The primary components of the prototype and its design are detailed below.

The presented camera prototype contains four individual spectral channels conjugated with a single IS (DMK 33GX264, IMX264 2/3″ sensor, 2448 × 2048 pixels, 3.45 μm pixel size, The Imaging Source, Bremen, Germany). The identical objectives with 75 mm focal length and F#2.8 (VM7528MP5, Guangdong ZLKC Optical Co., Ltd., Guangzhou, China) are equipped with 1″ bandpass spectral filters. Central wavelengths (CWL) and the full width at half maximum (FWHM) of filters’ spectral bands are shown in Table 1.

**Figure 1:**
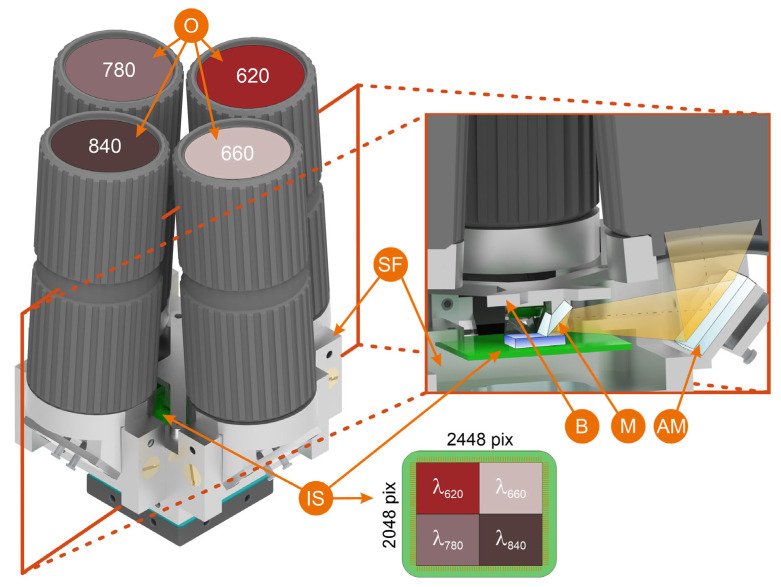
MS camera layout and ray tracing for a single spectral band: O—objectives with mounted bandpass filters; SF—supporting frame assembly; IS—image sensor; AM—adjustable mirrors; M—stationary mirrors; B—stationary mirrors base.

**Figure 2:**
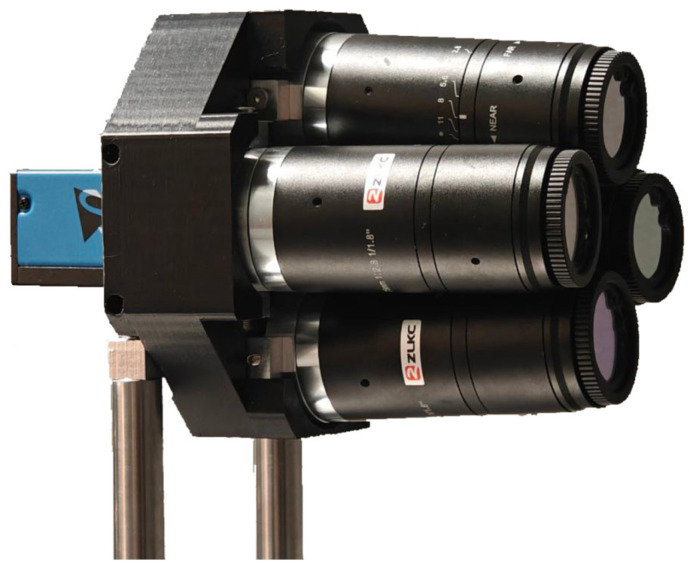
The assembled MS camera prototype.

Figure 1 illustrates the MS camera layout configuration. The supporting frame assembly SF includes the frame itself and four adjustable pivots for C-mount objectives O and adjustable mirrors AM. The optical axis direction for each individual spectral channel is altered twice using a combination of adjustable and fixed mirrors. Fixed mirrors M are mounted on base B in front of the image sensor IS. To correct for position deviations in the fixed mirrors, we incorporated the capability to finely adjust the position of the adjustable mirrors while keeping the pivots stationary. To minimize stray light, we enclosed the support frame assembly in a custom-made opaque housing. The completed MS camera prototype is depicted in Figure 2.

#### 2.1.2. Auxiliary Equipment and Calibration Targets

**Figure 3:**
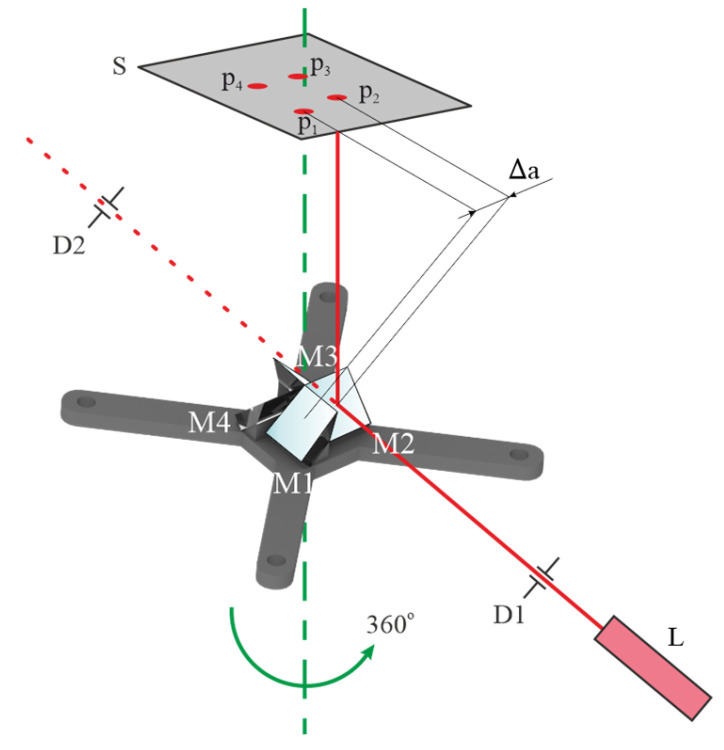
Setup for mirror alignment and displacement control.

Since the stationary mirrors are near the IS to minimize the distance between the objectives and the IS, they need to be very compact. The ones in the proposed MS camera prototype are attached to the base with glue. To ensure proper alignment during assembly and evaluate displacement due to glue shrinkage we implemented an auxiliary laser-based setup (Figure 3). The base with attached mirrors is located on a precise rotating stage. Prior to this, the laser O beam axis is aligned with the pair of irises D1, D2. Direction angles for each mirror are determined using the position deviations ${∆a}_{i}$ of laser spot positions p_1_–p_4_ observed on a screen S and a distance to the screen. If the optimal alignment is achieved, ∆a = 0. In practice, the determined position deviations may be accounted for during fine alignment of adjustable mirrors. Finally, to overcome uncompensated image rotation and achieve point-to-point correspondence of images obtained with individual spectral channels, we applied camera calibration described in Section 2.2.

We determined the spatial image transformations associated with correction of spatial distortions using the images of a checkerboard pattern test target. To quantify the dependence of the sensor response on exposure time, a reference source (integrating sphere) with stable radiative output was employed (Spectra-FT-2300-W, Labsphere Inc., North Sutton, NH, USA). The integrating sphere was also implemented for vignetting correction. The spectral response of individual spectral channels was assessed involving images of a diffuse scattering reference target providing uniform reflectance close to 1 across the relevant spectral range illuminated with the tunable light source (broadband light source: XWS-65, ISTEQ, Eindhoven, The Netherlands; monochromator: M266i–IV, Solar Laser Systems, Minsk, Republic of Belarus). Temperature mapping validation required a blackbody source (Metropir Helios, NPL Metropir LLC, Saint Petersburg, Russia, temperature range 800–1500 °C).

### 2.2. Camera Calibration

**Figure 4:**
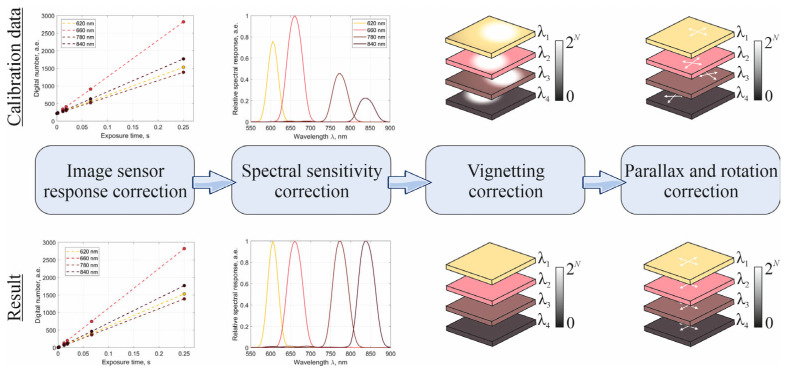
Main stages of spatial–spectral distortion correction of MS data.

The MS data captured by the camera can be distorted due to variations in the actual transmission bands of spectral filters, the optical system characteristics, and the IS response and sensitivity. Each of these factors impacts the resulting spectral curve of the observed object and subsequently reduces the accuracy of temperature estimation. To mitigate this, proper calibration of the MS camera is essential. This process entails calculating correction coefficients for each spectral channel and applying them to adjust the raw data. The primary stages of the correction workflow, developed using reference targets and calibrated laboratory radiation sources, are depicted in Figure 4.

The IS response describes the relationship between the radiant flux and the pixel intensity values. Distortions caused by the non-linearity and disproportionality of the light-sensitive element and electronic components [28] introduce systematic errors that reduce the accuracy of spectral measurements, narrow the dynamic range and complicate the comparison of data acquired at different times or using different devices. Several studies [29,30] have examined sensor response functions and how they vary with changes in light intensity and exposure time. Due to the pronounced curvature of the Planck distribution in the 550–900 nm spectral range, image acquisition can be performed across a range of exposure times, either for each spectral channel or for different temperature regimes. An integrating sphere was used to provide spatially uniform, broadband illumination across the MS camera’s field of view, allowing us to measure how the IS response changes with exposure time. We acquired a series of images of the sphere’s output port at different exposure settings and normalized the results. This allowed calculating correction coefficients that make the sensor output directly proportional to the incoming radiance. Applying these corrections enables automatic exposure adjustment during real-time monitoring.

The spectral sensitivity of the MS camera is determined by both the sensor’s sensitivity and the transmission characteristics of the spectral filters in each optical channel. These properties are typically assumed to be spatially uniform. Spectral non-uniformities can distort the shape of an object’s spectral signature. It becomes especially critical in spectroscopic applications, such as spectral thermal imaging. To quantify and correct these sensitivity variations, diffuse scattering reference target images were captured. By averaging the signal across the image and accounting for both the measured radiation power and the sensor’s response, we derived the camera’s sensitivity curves (shown in Figure 4). Correction coefficients were then calculated by integrating under these curves, allowing us to equalize the sensitivity across all channels and ensure consistent spectral measurements.

Optical system vignetting causes a gradual reduction in irradiance from the center of the image toward its boundaries. This spatial non-uniformity introduces systematic errors when reconstructing temperature maps, since accurate temperature measurements depend on precise pixel-level intensity values. Due to vignetting, calculated thermal gradients (like those found in melt pools) can be distorted, potentially leading to incorrect conclusions about the object’s thermal state. To correct for vignetting, the method described in [31] can be used. It involves capturing images of a spatially uniform radiance source (an integrating sphere) with the MS camera at various intensity levels. By averaging these images and normalizing them to the highest value, a correction matrix is derived. This matrix compensates for vignetting by adjusting the measured data, restoring radiometric uniformity across the entire field of view.

The proposed realization of a finite-conjugate MAFC introduces parallax—a shift in the position of objects between images from different channels due to differences in the optical path geometry. This issue is especially important when precise pixel-level alignment is needed for accurate mapping of physical parameters like temperature. Additional distortions come from the mirror system in the MS camera design, which can cause angular rotation of the images in individual channels. These effects cause spatial misalignment of objects across the spectral images, making it impossible to directly use the data for quantitative analysis, including temperature mapping, without first correcting the geometric distortions. To address this, we captured images of a checkerboard pattern test target using the MS camera. Matching points on the target were manually identified in each image, and rotation and translation matrices [32] were calculated for each channel relative to a reference channel. The 660 nm channel was chosen as the reference because its image center was closest to the center of the corresponding sensor area.

The algorithms for determining correction coefficients and transformation matrices, along with the data correction procedures, were implemented in Mathworks MATLAB R2024a.

### 2.3. Temperature Calculation

The application of correction coefficients and transformation matrices to the spectral images allows us to build a three-dimensional MS data cube, where each pixel represents the brightness of a corresponding area on the emitting object. Several methods can then be used to estimate temperature from the emission spectrum: the colorimetric method [33], Wien’s displacement law [13], TWIP [10], and fitting the experimental spectrum to the blackbody radiation curve [34].

The colorimetric method, which estimates the object’s color temperature, requires only an RGB camera, offering a simple and cost-effective solution. However, this method yields a subjective parameter influenced by both the spectral composition of the emission and the characteristics of the standard observer, resulting in a measured color temperature that may not correspond precisely to the actual thermodynamic temperature. Additionally, it is sensitive to spectral distortions introduced by sensor characteristics, environmental conditions, and color processing algorithms. Wien’s displacement law is applicable only within a temperature range of approximately 3000 to 7000 °C, where the emission peak lies within the visible spectrum; outside this range, it is only useful as a rough estimate.

TWIP requires brightness measurements in only two spectral bands and is robust against partial spectral distortions because it relies on brightness ratios. However, at lower temperatures (around 1000 to 2000 °C), it can produce significant errors due to the small differences in intensity (i.e., the low logarithmic ratio of intensities). Since the camera we developed has four spectral channels, the two-color pyrometry method can be used to provide initial conditions for the blackbody radiation fitting.

The approximation method ensures both versatility and high precision in temperature estimation. While the high quantity of spectral bands is ideal for accurate approximation, it can still be effectively applied using only four spectral channels, provided these channels are evenly distributed across the spectrum to adequately capture the characteristic shape of the Planck radiation curve. In this context, the method reduces to fitting four measured intensity values to the theoretical curve by optimizing the temperature parameter T along with the scaling coefficient ε:

(3) $$\sum_{i=1}^{4}{\left(I(\lambda_{i})-\varepsilon \cdot I^{\prime }(\lambda_{i},T)\right)}^{2}\to min,$$

where $I(\lambda_{i})$ represents the emission spectrum acquired by the MS camera, averaged either over a specified region or at an individual pixel, while $I^{\prime }(\lambda_{i},T)$ represents the theoretical Planck radiation curve, with i indexing the spectral channels. Optimization of the temperature parameter and scaling factor was performed using the Lagarias simplex search algorithm [35], utilizing MATLAB R2024a built-in optimization functions. After optimization, the temperature was converted from Kelvin to Celsius. Initial parameter estimates for the optimization were derived from two-color pyrometry calculations based on the 780 nm and 840 nm spectral channels. This pixel-wise optimization of MS data facilitates high-resolution spatial mapping of the temperature distribution across the observed object.

Emissivity ε is a parameter that determines the radiation intensity in comparison to an ideal blackbody. During the optimization process the emissivity value is varied along with the temperature to minimize the difference between the theoretical radiation and the actual measured MS data. In practice, when studying processes with known materials, approximate tabulated emissivity values are used [36,37]. Our optimization algorithm allows setting the initial emissivity range guess from tabulated values. This reduces the complexity of the problem, making the calculations faster and improving the stability of the optimization. For the development of an operational temperature estimation algorithm, we applied this approximation.

## 3. Results

### 3.1. Blackbody Cavity Validation

To validate the proposed methodology and quantify its accuracy, imaging was performed on the output aperture of a temperature-controlled blackbody source. The temperature was systematically varied over the range ∆T = 900 to 1400 °C in 50 °C increments. After each temperature adjustment, a stabilization time interval was observed, as indicated by the instrument’s status indicator. Exposure times at each step were optimized to maintain the maximum signal level at approximately 70% of the sensor’s dynamic range, avoiding non-linear response regions of the sensor. Due to the angular dependence of the blackbody source emission characteristics [38], off-axis imaging can introduce spectral distortions that violate the underlying assumptions of the radiometric model. To mitigate these effects, optomechanical components allowed precise adjustment of the MS camera’s orientation and lateral positioning along axes both parallel and perpendicular to the blackbody aperture plane. Through iterative angular and translational alignment for each spectral channel, the emitting source was maintained on the optical axis of the imaging system throughout data acquisition.

**Figure 5:**
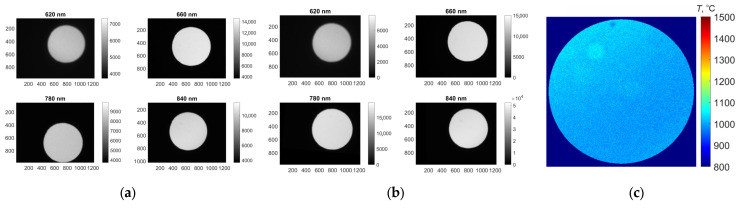
(**a**) Raw images of the blackbody source output aperture; (**b**) Corrected spectral images; (**c**) Temperature map of the blackbody source (T = 1000 °C).

Figure 5 presents the raw MS images of the blackbody aperture, the spectral data after distortion correction, and an example of the resulting spatial temperature distribution.

**Figure 6:**
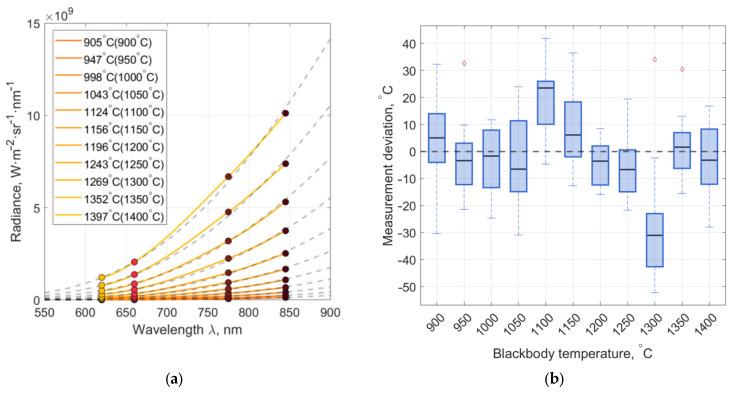
(**a**) Fitting results for averaged MS data (solid) and the theoretical Planck curve (dashed) for 11 blackbody temperatures; (**b**) Deviations of the temperature averaged over the output aperture from the true temperature.

During data processing, it was observed that the approximation method becomes unstable when limited spectral channels are used. This is reflected in the scatter of temperature estimates across repeated calculations, indicating the presence of multiple local minima in the error function of the non-linear optimization. To assess the method’s stability and quantify potential errors, the data were processed 15 times, followed by an analysis of the mean values and standard deviations of the temperature error. Temperature estimation was based on MS data averaged over the output aperture area. The results are shown in Figure 6.

The mean absolute error was 8 °C, the maximum absolute error 31 °C, the mean relative error 1%, and the standard deviation of the relative error 0.78% for the selected temperature range.

### 3.2. Tungsten Lamp

To validate the capability of temperature mapping under non-uniform thermal conditions, spectral imaging was performed on a tungsten filament lamp (TRU-1100-2300) [39] containing structural defects. This lamp model has a rated service life of 50 h at a maximum current of 30 A and a maximum filament temperature of 2350 °C. For this study, we examined a laboratory lamp that had far surpassed its expected lifespan. We utilized this overaged lamp to identify any potential defects that may have developed. When the service life of the tungsten heating element is exceeded, it begins to degrade due to grain growth in the metal, resulting in significant changes to the metal’s surface and, consequently, its emissivity.

**Figure 7:**
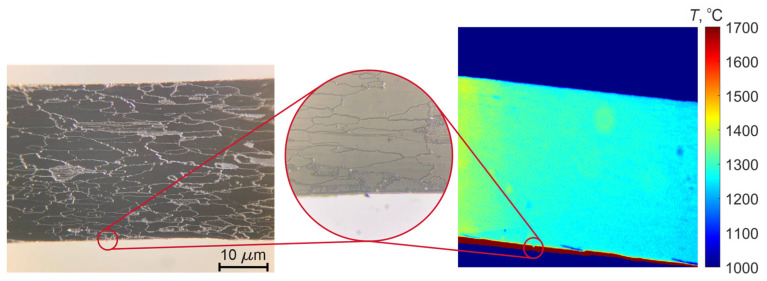
Surface morphology and thermal distribution of the lamp filament.

Figure 7 displays the surface of the filament ribbon and its distinct intergranular structure. The brightfield image was captured using a metallographic microscope in the same area where the MS camera acquired images. A burr-shaped defect was selected as a reference point; it is clearly visible under the microscope both when the lamp is turned off and at elevated temperatures during the experiment. The object temperature exhibits a non-linear dependence on applied current and voltage. Consequently, temperature maps were acquired by varying the current from 9 A to 16 A in 2 A increments and the voltage from 1.5 V to 4 V in 0.1 V increments. At each measurement point, the exposure time was calibrated to maintain the maximum signal level at approximately 70% of the detector’s dynamic range to avoid non-linear response. The MS camera IS plane was aligned parallel to the emitting surface at a fixed distance.

**Figure 8:**
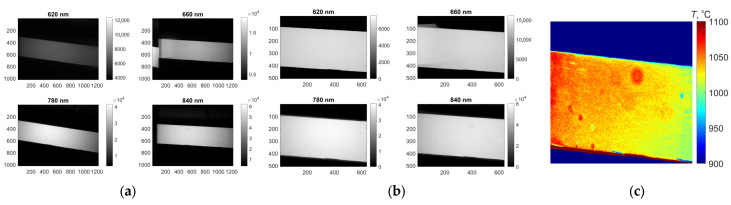
(**a**) Raw images of the lamp; (**b**) Corrected spectral images; (**c**) Lamp temperature map.

Figure 8 displays the raw output images of the lamp, the spectral images after geometric and radiometric corrections, and a representative reconstructed temperature distribution map.

**Figure 9:**
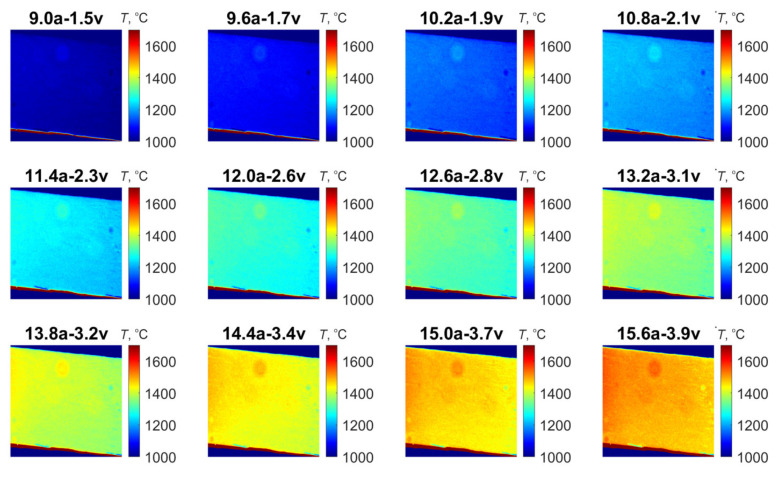
Temperature distribution maps of the lamp at various power settings.

The raw image from the 660 nm channel (Figure 8a) displays noticeable reflections related to stationary mirrors’ base construction flaw, necessitating cropping of the final temperature map to a resolution of 456 × 639 pixels. To prevent unwanted image intersection in spectral channels, the stationary mirrors’ base should include non-reflecting blinds that prevent inter-channel reflections. Figure 9 shows the temperature distribution maps obtained at various lamp power settings.

## 4. Discussion

Experimental validation highlighted potential advantages and drawbacks of the proposed approach. Although the image resolution decreases due to IS division and cropping during the correction stage, the resulting temperature maps still provide sufficient detail to identify defects and local temperature variations. Differences in temperature values at similar power levels, compared to the results described in [39], are related to filament degradation and non-uniform filament heating. The spatial offset in the image acquisition area relative to the filament center emphasizes the temperature gradient inherent to this object type at moderate power levels. The experimental technique used in [39] is based on combining a brightness imaging pyrometer that acquires images in a narrow band within the visible wavelength range with a point-measurement spectrometer. In this case, temperature mapping is achieved with a significant assumption of a graybody with a uniform emissivity spatial distribution. Detailed spectral intensity measurement provides precise temperature estimation at one point, and the temperature map is obtained by comparing the brightness in distinct pixels. A larger temperature error obtained with four-wavelength fitting compared to the values reported in [39] is associated with reduced spectral information relative to the point-measurement spectrometer. Similarly, line-scanning hyperspectral imaging approaches [20,21] demonstrate better temperature precision along with lower temporal resolution. Our prototype acquired 24 fps for 16-bit at full IS resolution with a single frame exposure time of less than 10 milliseconds. The data acquisition speed was limited by the bandwidth connection of the gigabit Ethernet interface. Using the proposed approach, one can achieve higher temporal resolution while monitoring non-stationary objects by selecting a faster IS and limiting the temperature precision.

The mentioned graybody assumption is widespread in common visible and IR imaging pyrometry techniques [13], limiting the attainable precision in real “non-gray” objects. To address the emissivity dependencies issue, one can refer to published data to obtain an initial estimate [40,41] and assume the simplified model [13,23]. However, within the context of the defined objectives, this approximation provides a reasonable balance between computational efficiency and required accuracy. The assumption of a known emissivity can lead to inaccuracies, particularly when the material’s surface properties evolve during heating [42]. The proposed approach allows pixel-wise emissivity fitting along with temperature, although it is time-consuming. To enhance fitting stability and provide the algorithm with an initial guess we introduced an ability to insert a set of characteristic emissivity ranges prior to temperature calculation. Thus, the proposed approach relies on the assumption of a finite set of material types in the field of view during the HED process. Also, using the proposed approach one can choose two of the four individual spectral images as TWIP data to achieve fast temperature estimation with potentially lower precision.

Implementation of four different spectral bands provides the researcher with an ability to implement the desired Planck’s curve fitting methods and improve calculation stability. The analysis of the coefficients of variation demonstrates a moderate degree of variability in the temperature estimates derived by proposed method. At lower temperatures, the coefficients range from 1.13 to 1.36, indicating higher measurement instability and greater fluctuations. As the temperature increases, the coefficients decrease (coefficients drop below 1, from 0.66 to 0.92), suggesting improved precision and reduced variability in the measurements at elevated temperatures. This trend may reflect enhanced measurement accuracy or a reduced sensitivity to external factors at higher temperature levels. To maintain the measurement accuracy at lower temperatures using the proposed approach, one can select bigger central wavelength values of spectral filters and extend the working spectral range in near IR by choosing the InGaAs-based IS if needed. Although reducing the accessibility, an interchangeable filter–IS combination may be achieved without objective replacement by application of broadband apochromatic optical systems [43,44,45]. In addition to simple temperature-regime-dependent reconfiguration, the flexible choice of filter transmission bands provides potential for avoiding the absorption bands of gases and evaporation plume [13,19] in HED industrial processes.

Another issue to consider is the angular variations in thermal radiation intensity with respect to the observation direction. The expected location of the thermal imaging device results in off-axis image acquisition. Additionally, the proposed camera design implies individual spectral channel parallax, leading to slightly different observation directions. Thus, the angle between the observation direction of each channel and the surface normal also needs to be considered. Since the emissivity is reported to remain constant in the range of angles between 0° and 40° [46], the channel directions should not exceed this range to avoid distortions. Assuming the heated object to be a Lambertian emitting surface the intensity variations in different off-axis directions of spectral channels may be taken into account. The alternative solution to minimize angular variations is implementation of a single-objective system based on spectrally resolving detector arrays [24] with Bayer-type filter arrays. Providing a spatial resolution decrease proportional to the number of spectral channels similar to the MAFC, this approach involves a technologically complex commercially available IS with embedded fixed patterns of spectral filters, significantly increasing the cost relative to a conventional monochrome IS and reducing the flexibility. Since the optimal spectral channel bandwidth is a tradeoff between light throughput and spectral resolution, overlapping spectral bands of Bayer-type filters lead to channel cross-talk identical to RGB cameras that require additional correction [47].

It is also important to note the complex impact of parallax in the optical system. During the development of our MS camera, we intentionally minimized parallax to mitigate potential distortions caused by differences in viewing angles. However, accounting for the dependence of thermal radiation intensity on the viewing angle [46], we can increase both the parallax and the stereoscopic baseline (while adhering to the constraints on lens-to-sensor distance) to enable multispectral stereo functionality [48]. This approach enhances spectral measurement devices by adding the capability to reconstruct the 3D shape of the observed object’s surface.

## 5. Conclusions

Experimental validation of the prototype developed using the basic design concept and data processing workflow proposed in this work demonstrated competitive temperature uncertainty compared to other thermal imaging techniques, while maintaining the potential for quick and simple reconfiguration to adapt to object parameters (scale, working distance, temperature regime). The primary considerations of the MS MAFC basic design concept, including geometric optics assumptions and alignment recommendations, are presented. The correction workflow enables one to assemble a camera using commercially available components and personally assess and account for their characteristic properties, even without comprehensive datasheet specifications.

Future research will focus on two main aspects: (1) further development of the basic design concept to elucidate the rationale behind the optimal selection of spectral channels’ central wavelengths and bandwidths; (2) conducting in situ experiments to evaluate the effectiveness and practicality of the proposed MS camera design and workflow for HED industrial processes and AM monitoring using the prototype. We will also optimize algorithms to enable real-time temperature mapping.

## Figures and Tables

**Table 1:** Characteristics of the implemented bandpass filters.

CWL, nm	FWHM, nm
620	30
660	40
780	42
840	45

## Data Availability

Experimental data are available from the corresponding author upon reasonable request.

## Abbreviations

The following abbreviations are used in this manuscript:

AM	Additive manufacturing
CWL	Central wavelength
FWHM	Full width at half maximum
HED	High energy density
IS	Image sensor
IR	Infrared wavelength range
MAFC	Multiaperture filtered camera
MS	Multispectral
TWIP	Two-wavelength imaging pyrometry

## References

[1] Das M., Dixit U.S., Davim J.P. (2018). Advanced Machining Processes. Introduction to Mechanical Engineering.

[2] Patterson T., Hochanadel J., Sutton S., Panton B., Lippold J. (2021). A review of high energy density beam processes for welding and additive manufacturing applications. Weld. World.

[3] Najmon J.C., Raeisi S., Tovar A., Froes F., Boyer R. (2019). Review of additive manufacturing technologies and applications in the aerospace industry. Additive Manufacturing for the Aerospace Industry.

[4] Osipovich K., Kalashnikov K., Chumaevskii A., Gurianov D., Kalashnikova T., Vorontsov A., Zykova A., Utyaganova V., Panfilov A., Nikolaeva A. (2023). Wire-Feed Electron Beam Additive Manufacturing: A Review. Metals.

[5] Razavykia A., Brusa E., Delprete C., Yavari R. (2020). An Overview of Additive Manufacturing Technologies—A Review to Technical Synthesis in Numerical Study of Selective Laser Melting. Materials.

[6] Vafadar A., Guzzomi F., Rassau A., Hayward K. (2021). Advances in Metal Additive Manufacturing: A Review of Common Processes, Industrial Applications, and Current Challenges. Appl. Sci..

[7] Chen Z., Han C., Gao M., Kandukuri S.Y., Zhou K. (2022). A review on qualification and certification for metal additive manufacturing. Virtual Phys. Prototyp..

[8] Yoshioka J., Eshraghi M. (2023). Temporal evolution of temperature gradient and solidification rate in laser powder bed fusion additive manufacturing. Heat Mass Transf..

[9] Mamuschkin V., Haeusler A., Engelmann C., Olowinsky A., Aehling H. (2017). Enabling pyrometry in absorber-free laser transmission welding through pulsed irradiation. J. Laser Appl..

[10] Belikov R., Merges D., Varentsov D., Major Z., Neumayer P., Hesselbach P., Schanz M., Winkler B. (2024). Fast Multi-Wavelength Pyrometer for Dynamic Temperature Measurements. Int. J. Thermophys..

[11] Vuelban E.M., Girard F., Battuello M., Nemeček P., Maniur M., Pavlásek P., Paans T. (2015). Radiometric Techniques for Emissivity and Temperature Measurements for Industrial Applications. Int. J. Thermophys..

[12] Everton S.K., Hirsch M., Stravroulakis P., Leach R.K., Clare A.T. (2016). Review of in-situ process monitoring and in-situ metrology for metal additive manufacturing. Mater. Des..

[13] Grujić K. (2023). A Review of Thermal Spectral Imaging Methods for Monitoring High-Temperature Molten Material Streams. Sensors.

[14] Rodriguez E., Mireles J., Terrazas C.A., Espalin D., Perez M.A., Wicker R.B. (2015). Approximation of absolute surface temperature measurements of powder bed fusion additive manufacturing technology using in situ infrared thermography. Addit. Manuf..

[15] Mazzarisi M., Angelastro A., Latte M., Colucci T., Palano F., Campanelli S.L. (2023). Thermal monitoring of laser metal deposition strategies using infrared thermography. J. Manuf. Process..

[16] Haley J., Karandikar J., Herberger C., MacDonald E., Feldhausen T., Lee Y. (2024). Review of in situ process monitoring for metal hybrid directed energy deposition. J. Manuf. Process..

[17] Ding X.P., Li H.M., Zhu J.Q., Wang G.Y., Cao H.Z., Zhang Q., Ma H.L. (2017). Application of infrared thermography for laser metal-wire additive manufacturing in vacuum. Infrared Phys. Technol..

[18] Vallabh C.K.P., Zhao X. (2022). Melt pool temperature measurement and monitoring during laser powder bed fusion based additive manufacturing via single-camera two-wavelength imaging pyrometry (STWIP). J. Manuf. Process..

[19] Myers A.J., Quirarte G., Ogoke F., Lane B.M., Uddin S.Z., Farimani A.B., Beuth J.L., Malen J.A. (2023). High-resolution melt pool thermal imaging for metals additive manufacturing using the two-color method with a color camera. Addit. Manuf..

[20] Devesse W., De Baere D., Guillaume P. (2017). High Resolution Temperature Measurement of Liquid Stainless Steel Using Hyperspectral Imaging. Sensors.

[21] Staudt T., Eschner E., Schmidt M. (2019). Temperature determination in laser welding based upon a hyperspectral imaging technique. CIRP Ann..

[22] Poissenot-Arrigoni C., Marcon B., Rossi F., Fromentin G. (2023). In-Situ Pixel-Wise Emissivity Measurement Using a Multispectral Infrared Camera. J. Imaging.

[23] Qu D.-X., Berry J., Calta N.P., Crumb M.F., Guss G., Matthews M.J. (2020). Temperature Measurement of Laser-Irradiated Metals Using Hyperspectral Imaging. Phys. Rev. Appl..

[24] Hagen N., Kudenov M. (2013). Review of snapshot spectral imaging technologies. Opt. Eng..

[25] Shogenji R., Kitamura Y., Yamada K., Miyatake S., Tanida J. (2004). Multispectral imaging using compact compound optics. Opt. Express.

[26] Batshev V.I., Krioukov A.V., Machikhin A.S., Zolotukhina A.A. (2023). Multispectral video camera optical system. J. Opt. Technol..

[27] Malacara D., Malacara Z. (2004). Handbook of Optical Design.

[28] Nehir M., Frank C., Aßmann S., Achterberg E.P. (2019). Improving Optical Measurements: Non-Linearity Compensation of Compact Charge-Coupled Device (CCD) Spectrometers. Sensors.

[29] Chen C., Mccloskey S., Yu J. Analyzing Modern Camera Response Functions. Proceedings of the 2019 IEEE Winter Conference on Applications of Computer Vision (WACV).

[30] Tai Y.W., Chen X., Kim S., Kim S.J., Li F., Yang J., Yu J., Matsushita Y., Brown M.S. (2013). Nonlinear camera response functions and image deblurring: Theoretical analysis and practice. IEEE Trans. Pattern. Anal. Mach. Intell..

[31] Cao H., Gu X., Wei X., Yu T., Zhang H. (2020). Lookup Table Approach for Radiometric Calibration of Miniaturized Multispectral Camera Mounted on an Unmanned Aerial Vehicle. Remote Sens..

[32] Muthukumaran D., Sivakumar M. (2017). Medical Image Registration: A Matlab Based Approach. Int. J. Sci. Res. Comput. Sci. Eng. Inf. Technol..

[33] Li C., Kong D., Wang Y., Gao L., Zhang X., Zhang Q. (2023). Color CCD High-Temperature Measurement Method Based on Matrix Searching. Appl. Sci..

[34] Andreić Ž. (1992). Distribution temperature calculations by fitting the Planck radiation curve to a measured spectrum. Appl. Opt..

[35] Lagarias J.C., Reeds J.A., Wright M.H., Wright P.E. (1998). Convergence Properties of the Nelder--Mead Simplex Method in Low Dimensions. SIAM J. Optim..

[36] Haynes W.M. (2016). Handbook of Chemistry and Physics.

[37] Goldswith A., Waterman T.E., Hirschhorn H.J. (1961). Handbook of Thermophysical Properties of Solid Materials.

[38] Nester A., Mahan J.R. (2002). Spatial and angular distributions for irradiance from blackbody cavities. Proc. SPIE.

[39] Gulyaev I.P., Dolmatov A.V. (2018). Spectral-brightness pyrometry: Radiometric measurements of non-uniform temperature distributions. Int. J. Heat Mass Transf..

[40] Kobayashi M., Ono A., Otsuki M., Sakate H., Sakuma F. (1999). A Database of Normal Spectral Emissivities of Metals at High Temperatures. Int. J. Thermophys..

[41] Baibakova V., Elzouka M., Lubner S., Prasher R., Jain A. (2022). Optical emissivity dataset of multi-material heterogeneous designs generated with automated figure extraction. Sci. Data.

[42] Setién-Fernández I., Echániz T., González-Fernández L., Pérez-Sáez R.B., Tello M.J. (2014). Spectral emissivity of copper and nickel in the mid-infrared range between 250 and 900 °C. Int. J. Heat Mass Transf..

[43] Lu Q., Ding Y., Wang W., Liu S., Xu M. (2022). VIS-NIR superachromatic triplet design with five-color correction for abroadband interferometer. Appl. Opt..

[44] Poliakov M.P., Khokhlov D.D., Bykov A.A. (2025). Apochromatic objective for imaging spectral systems of visible, near and short-wave infrared spectrum ranges. Sci. Tech. J. Inf. Technol. Mech. Opt..

[45] ViSWIR Lens Series—Computar. https://www.computar.com/series/viswir.

[46] Incropera F.P., Dewitt D.P., Bergman T.L., Lavine A.S. (2010). Fundamentals of Heat and Mass Transfer.

[47] Kim J., Jeong K., Kang M.G. (2022). Crosstalk Correction for Color Filter Array Image Sensors Based on Lp-Regularized Multi-Channel Deconvolution. Sensors.

[48] Wisotzky E.L., Triller J., Kossack B., Globke B., Arens P., Hilsmann A., Eisert P. (2023). From Multispectral-Stereo to Intraoperative Hyperspectral Imaging: A Feasibility Study. Curr. Dir. Biomed. Eng..

